# Muscle Stem/Progenitor Cells and Mesenchymal Stem Cells of Bone Marrow Origin for Skeletal Muscle Regeneration in Muscular Dystrophies

**DOI:** 10.1007/s00005-018-0509-7

**Published:** 2018-03-13

**Authors:** Aleksandra Klimczak, Urszula Kozlowska, Maciej Kurpisz

**Affiliations:** 1 Hirszfeld Institute of Immunology and Experimental Therapy, Polish Academy of Sciences, Wroclaw, Poland; 2 Institute of Human Genetics, Polish Academy of Sciences, Poznan, Poland

**Keywords:** Muscle stem/progenitor cells, Mesenchymal stem cells, Skeletal muscle regeneration, Muscular dystrophies

## Abstract

Muscular dystrophies represent a group of diseases which may develop in several forms, and severity of the disease is usually associated with gene mutations. In skeletal muscle regeneration and in muscular dystrophies, both innate and adaptive immune responses are involved. The regenerative potential of mesenchymal stem/stromal cells (MSCs) of bone marrow origin was confirmed by the ability to differentiate into diverse tissues and by their immunomodulatory and anti-inflammatory properties by secretion of a variety of growth factors and anti-inflammatory cytokines. Skeletal muscle comprises different types of stem/progenitor cells such as satellite cells and non-satellite stem cells including MSCs, interstitial stem cells positive for stress mediator PW1 expression and negative for PAX7 called PICs (PW1^+^/PAX7^−^ interstitial cells), fibro/adipogenic progenitors/mesenchymal stem cells, muscle side population cells and muscle resident pericytes, and all of them actively participate in the muscle regeneration process. In this review, we present biological properties of MSCs of bone marrow origin and a heterogeneous population of muscle-resident stem/progenitor cells, their interaction with the inflammatory environment of dystrophic muscle and potential implications for cellular therapies for muscle regeneration. Subsequently, we propose—based on current research results, conclusions, and our own experience—hypothetical mechanisms for modulation of the complete muscle regeneration process to treat muscular dystrophies.

## Introduction

Skeletal muscle is post-mitotic tissue capable of repair and regeneration after injury. Stimulus or damage of skeletal muscle arising from physiological conditions (exercise, aging) or diseases (cachexia, sarcopenia, muscular dystrophies) triggers the regenerative process. In the regenerative process of skeletal muscle, different types of cells are involved, including muscle-resident progenitors and cells involved in innate and adaptive immune responses (Judson et al. 2013; Madaro and Bouche 2014). The regenerative potential of skeletal muscle is maintained by the heterogeneous population of muscle-resident stem/progenitor cells including satellite cells (SCs) capable of regenerating muscle fibers and maintaining a functional satellite pool, and by non-satellite stem cells: mesenchymal stem/stromal cells (MSCs), PW1^+^/PAX7^−^ interstitial cells (PICs), fibro/adipogenic progenitors/mesenchymal stem cells (FAPs/MSCs), muscle side population (SP) cells and muscle resident pericytes. In response to an injury signal, myogenic SCs become activated, release chemotactic factors and a panel of pro-inflammatory cytokines, and attract monocytes and macrophages to the injury site. Activated SCs proliferate, migrate from their niche to the injury site, and generate myoblasts, which either fuse to damaged myofibers or fuse together to form myotubes that mature and form new muscle fibers. However, myogenic activity of SCs is supported by a heterogeneous population of muscle-resident MSCs which contribute to skeletal muscle regeneration (Joe et al. 2010; Judson et al. 2013; Uezumi et al. 2011). Direct contact of SCs with immune cells, especially those responsible for innate immunity, permits proper muscle regeneration. In adult human muscle macrophages orchestrate myogenesis and muscle regeneration by the interactions of differentially activated macrophages with SCs. Studies performed in vitro on human SCs and macrophages documented that pro-inflammatory macrophages (M1) inhibited myogenic precursor cell (MPC) fusion while anti-inflammatory macrophages (M2) strongly promoted MPC differentiation by increasing their commitment to differentiated myoblasts and the formation of mature myotubes (Saclier et al. 2013). Dysregulation in cooperation between muscle progenitors and cells responsible for adaptive and innate immune responses leads to impaired muscle regeneration and deposition of non-functional adipose and fibrotic tissue as occurs in muscular dystrophies (Alexakis et al. 2007; Madaro and Bouche 2014).

Researchers worldwide are working on diverse strategies to create innovative therapies for injured and/or degenerated skeletal muscle of dystrophic or traumatic patients. But first, to make cellular therapies effective, we need to clearly understand the links between MSCs and other muscle regeneration progenitor cells and inflammatory cell systems in the process of muscle regeneration in vivo and in vitro. There is still a need to investigate and gain more information about this process, especially in the area of paracrine communications between cells. In this review, we propose—based on current research results, conclusions and own experience—to introduce biological properties of MSCs of bone marrow (BM) origin and the heterogeneous population of muscle-resident stem/progenitor cells, and subsequently hypothetical mechanisms modulating the complete muscle regeneration process to treat muscular dystrophies.

## Influence of Innate and Adaptive Immune Response in Muscular Dystrophies

Muscular dystrophies are a group of diseases which may develop in several forms, and severity of disease is associated with mutations in genes coding proteins associated with the muscle membrane, such as the dystrophin–glycoprotein complex (Hoffman et al. 1988; Matsumura and Campbell 1993), or with the extracellular matrix (ECM), such as laminin and collagen VI (Qiao et al. 2005; Zou et al. 2008). The absence of dystrophin (a protein linking cytoskeletal component) leads to increased fragility of the sarcolemma. Damage of muscle fibers leads to activation of SCs, responsible for muscle growth and regeneration, and induces an inflammatory response. The inflammatory response in damaged muscle is initiated due to the ability of SCs to secrete chemotactic factors such as monocyte chemotactic protein 1, macrophage-derived chemokine, fractalkine, urokinase-type plasminogen activator/urokinase-type plasminogen activator receptor (uPA/uPAR) and the vascular endothelial growth factor (VEGF) (Chazaud et al. 2003; Tidball and Villalta 2010).

Both innate and adaptive immune responses are involved in skeletal muscle regeneration in normal conditions and in muscular dystrophies. Acute injury of skeletal muscle triggers an innate immune response characterized by release of pro-inflammatory cytokines interleukin (IL)-1, IL-6 and tumor necrosis factor (TNF)-α at the site of injury. Myogenic precursor cells receive signals from injured muscle and attract monocytes from muscle-supplying vessels. Pro-inflammatory cytokines, especially interferon (IFN)-γ and TNF-α, transform monocytes into phagocytic M1 phenotype. M1 macrophages are important for pathogen prevention and for the phagocytosis of cellular debris, but they are not helpful in the muscle regeneration process because their ability to secrete a cytotoxic level of nitric oxide (NO) accelerates muscle injury (Villalta et al. 2009). A high concentration of M1 is associated with pro-inflammatory cytokine activity during the first step of muscle damage. This initial inflammatory response is diminished by anti-inflammatory Th2 cytokines, IL-4, IL-10 and IL-13, due to the switch of macrophage phenotype from M1 to M2. There are two pathways shifting macrophages from M1 to M2. The first is paracrine information delivered from Th2 lymphocytes by secretion of IL-4 and IL-13 facilitating transformation of M1 macrophage into M2a (CD206^+^) phenotype, and secretion of IL-10 leading to transformation of M1 into M2c (CD206^+^, CD163^+^) macrophages (Deng et al. 2012; Villalta et al. 2009). Both of them make an important contribution to the muscle regeneration process. M2a macrophages are known from supporting muscle regeneration, while M2c type suppresses activity of cytotoxic M1 macrophages and persists in damaged muscle until the termination of inflammation (reviewed by Tidball and Villalta 2010; Yin et al. 2013). There was also an assumption that phagocytic ability of M1 macrophages may contribute to shifting M1 phenotype into M2 phenotype. In vitro studies documented that after phagocytosis of dead muscle fiber debris M1 macrophages stopped secreting the inflammatory cytokine TNF-α and started to secrete transforming growth factor (TGF)-β, supportive for muscle regeneration, and this reflected the shift into M2 phenotype—that is the second mechanism in which this phenomenon occurs (Arnold et al. 2007). The switch of macrophage subsets is critical to muscle regeneration, as confirmed by depletion of monocytes/macrophages at different stages before and after muscle injury induced by a cardiotoxin in the mouse model (Wang et al. 2014). Moreover, M2 macrophages are able to suppress the inflammatory response and secrete paracrine factors fibroblast growth factor (FGF)-2, insulin growth factor (IGF)-1, IGF-2, hepatocyte growth factor (HGF), and IL-6 that support SCs activation, proliferation and differentiation, and additionally support neovascularization of new myofibers by platelet-derived growth factor (PDGF) secretion (Boonen and Post 2008; Tonkin et al. 2015). Macrophages also secrete the TGF-β family member, growth differentiation factor 3, which contributes to myoblast fusion (Varga et al. 2016). In situ transition of infiltrating macrophages from an inflammatory to a repair phenotype is dependent on the microenvironment and interaction with muscle progenitor cells as introduced in an acute sterile skeletal muscle injury mouse model (Patsalos et al. 2017).

Therefore, M2 macrophages and anti-inflammatory cytokines IL-4 and IL-10 reduce inflammation and contribute to satellite cell differentiation, thus promoting myogenic differentiation (Deng et al. 2012).

In the *mdx* mouse model, muscles are characterized by continuous cycles of myofiber necrosis and repair. Repetitive series of myofiber deterioration lead to muscle infiltration by M1 macrophages together with M2a macrophages, which may reduce cytotoxic activity of M1 macrophages (Villalta et al. 2009). The inflammatory environment in dystrophic muscle is comparable but not the same as in acute injury. Subsequent infiltration of M2c macrophages is associated with progression to the regenerative process. However, in acute injured muscle, the number of M2 macrophages decreases upon damage repair, while in mdx muscle their number increases with age and promotes fibrosis. Increased and persistent presence of macrophages modifies the microenvironment of dystrophic muscle, leading to amplified myofiber necrosis, and replacement of muscle with fibrotic and fat tissue.

In the mdx mouse, except neutrophils and macrophages, eosinophils play an important role in the innate immune response (Heredia et al. 2013; Madaro and Bouche 2014). Eosinophil invasion was found in Duchenne muscular dystrophy (DMD) patients and in mdx muscle, and was dependent on lymphocytes activity (Cai et al. 2000; Wehling-Henricks et al. 2008). In dystrophic muscle, eosinophils modulate injury and regeneration by promoting the transition from a Th1 to Th2 inflammatory environment. IL-4, the key cytokine produced by eosinophils, may support muscle regeneration; however, the primary targets of this cytokine are fibro-adipogenic progenitors (FAPs) (Heredia et al. 2013)—described below.

In normal steady-state conditions, lymphocytes are not involved in skeletal muscle regeneration, due to lack of ability of muscle fibers to induce a T-cell response as they do not express HLA class I or HLA class II antigens or co-stimulatory molecules (Karpati et al. 1988; Maffioletti et al. 2014). However, inducible expression of HLA class I and class II antigens on muscle fibers is generated in inflammatory muscle diseases. In this context, muscle cells act as nonprofessional antigen-presenting cells and attract T lymphocytes to the injury site and trigger a T-cell mediated immune response by modulation of the inflammatory cytokine milieu (Wiendl et al. 2003). Thus, the adaptive immune response is generally associated with chronic muscle dystrophies and persistence of T lymphocytes in dystrophic muscle exerts an influence on the muscle fiber environment and muscle cell function (Madaro and Bouche 2014; Spencer et al. 2001). However, the recruitment of regulatory T cells CD4^+^/CD25^+^/FOXP3^+^ to the injury site promotes muscle regeneration by direct contact with muscle precursor cells, as confirmed in a Rag2^−/−^ γ-chain^−/−^ mouse model (Castiglioni et al. 2015).

Thus, the immune response in muscular dystrophies introduced above in an experimental mdx mouse model and in clinical observations suggests that inflammation is considered as a feature of muscle repair and regulation of innate and adaptive immune responses may support muscle regeneration. This process may be supported by immunomodulatory activity of MSCs, which release anti-inflammatory factors and may create a favorable environment for muscle stem/progenitor cells for their differentiation and muscle repair.

## MSCs of Bone Marrow Origin

It is well known that MSCs in the BM environment constitute a part of the bone marrow stroma and create a specific niche supporting hematopoiesis (Klimczak and Kozlowska 2016; Majumdar et al. 1998). The regenerative potential of plastic-adherent stromal cells of BM origin was described for the first time by Friedenstein et al. (1966, 1974) by introducing their ability to regenerate or support ectopic bone, stroma and hematopoietic tissues. Further studies documented that MSCs have heterogeneous nature as they are linked to the development of various mesenchymal tissues. Caplan (1991) documented that an isolated adult bone marrow population of MSCs could give rise to a variety of tissues of mesenchymal origin by differentiating along separate and distinct lineage pathways. As they are associated with the formation of mesenchymal tissues during embryonic development, these cells were called MSCs (Caplan 1991). Subsequent studies performed by Caplan and co-workers, and other research groups, documented that MSCs are not only present in the BM compartment but relative abundance of MSCs was found throughout the body, and most of them are of perivascular origin (Caplan 2008; Caplan and Correa 2011; Crisan et al. 2008; da Silva Meirelles et al. 2009; Dellavalle et al. 2011).

Since that time extensive research on MSCs of BM origin has been performed to characterize their biology and surface epitopes. Heterogenicity of MSCs which reside in the human BM is exemplified by expression of a variety of surface epitopes including integrin receptors (CD29, CD49α), cell adhesion molecules (CD44, CD54, CD58, CD62L, CD105, CD106, CD146, CD166), enzymes (CD39, CD73), growth factor receptors (CD140b, CD271, CD340, CD349) intermediate filaments (nestin, vimentin, desmin, neurofilament) and embryonic antigens (SSEA-1), but none of these molecules have been specific for bone marrow-derived MSCs (Meirelles Lda and Nardi 2009; Rasini et al. 2013). The widespread capacities of MSCs in tissue repair are also accomplished by their ability to secrete a variety of bioactive proteins as part of their local trophic and immunomodulatory properties. MSCs are able to secrete growth factors and chemokines to induce proliferation and angiogenesis. Mitogenic factors produced by MSCs such as TGF-α, TGF-β, HGF, epithelial growth factor (EGF), basic FGF (FGF-2) and IGF induce epithelial and endothelial cell divisions. Moreover, IGF, EGF and VEGF secreted by MSCs may recruit endothelial precursor cells and initiate vascularization (Chen et al. 2008; Murphy et al. 2013).

The most interesting features of the biology of MSCs are their anti-inflammatory and immunomodulatory properties. The anti-inflammatory activity of MSCs is accomplished by their ability to secrete a variety of growth factors and anti-inflammatory cytokines affecting many types of immune cells including T cells, natural killer cells, B cells, monocytes, macrophages and dendritic cells. In response to inflammatory cytokines, such as IL-1, IL-2, IL-12, TNF-α, and IFN-γ, MSCs secrete a set of immunomodulatory factors including prostaglandin 2 (PGE2), TGF-β1, HGF, stromal-derived factor (SDF)-1, NO, indoleamine 2,3-dioxygenase, IL-4, IL-6, and IL-10, thereby limiting T-cell proliferation and function, and increasing T regulatory cell development and their activity (English et al. 2009; Miyagawa et al. 2017; Murphy et al. 2013). MSCs are also able to influence T-cell differentiation, and imbalance between Th1 and Th2 subpopulations of T lymphocytes in chronically inflamed microenvironments may be reversed by MSCs. MSCs promote transition of Th1 to Th2 type of T cells, thus reducing IFN-γ production by Th1 cells and increasing secretion of the more immunotolerant cytokines IL-4 and IL-10 by Th2 cells (Kong et al. 2009). Moreover, reduction of IFN-γ activity, and MSC-derived IL-4 and IL-10 have an influence on macrophages activity in inflamed tissue by conversion of macrophages from M1 (pro-inflammatory) to M2 (anti-inflammatory) (Murphy et al. 2013).

Superiority of MSCs as a therapeutic tool is due to the low or moderate expression of HLA class I antigens and lack or low expression of HLA class II antigens, making MSCs “invisible” to the recipient immune system in an allogeneic scenario. However, a pro-inflammatory environment and IFN-γ production may increase expression of their HLA class II antigens (Le Blanc et al. 2003; Siegel et al. 2009). Immunomodulatory activity of MSCs towards dendritic cells is associated with their capacity to produce anti-inflammatory factors (PGE2, TGF-β), which inhibit activation and maturation of dendritic cells, impairing their function. Crosstalk between MSCs and dendritic cells downregulates expression of co-stimulatory molecules (CD80, CD86), thus reducing the ability of dendritic cells to stimulate T cells (Nauta et al. 2006; Ramasamy et al. 2007).

Biological properties of MSCs provide an even wider tool with regenerative potential, as previously documented by their well-predicted therapeutic application in tissue regeneration of post-infarct heart (Jung et al. 2017; van den Akker et al. 2013). The immunomodulatory potential of MSCs is not only desired in the well-known therapy of graft-versus-host disease, and serious complications in patients after hematopoietic stem cell transplantation (Copland et al. 2015; Le Blanc et al. 2004; Lin and Hogan 2011), but also proved to be beneficial in therapy for skeletal, muscular (Maeda et al. 2017) and neural regeneration (Mokarram et al. 2012).

Summarizing the above data, MSCs of BM origin may have great curative potential in vivo due to their trophic, paracrine and immunomodulatory function. MSCs isolated from BM could be used as progenitors for tissue regeneration and tissue engineering to repair or replace damaged tissue of mesenchymal origin. Activated MSCs are not only able to differentiate into a specific cell lineage but may also establish a regenerative microenvironment by immunomodulatory activity regulating the local immune response.

## Skeletal Muscle Stem/Progenitor Cells

### Satellite Cells and Environmental Conditions in Muscle Regeneration

**Fig. 1:**
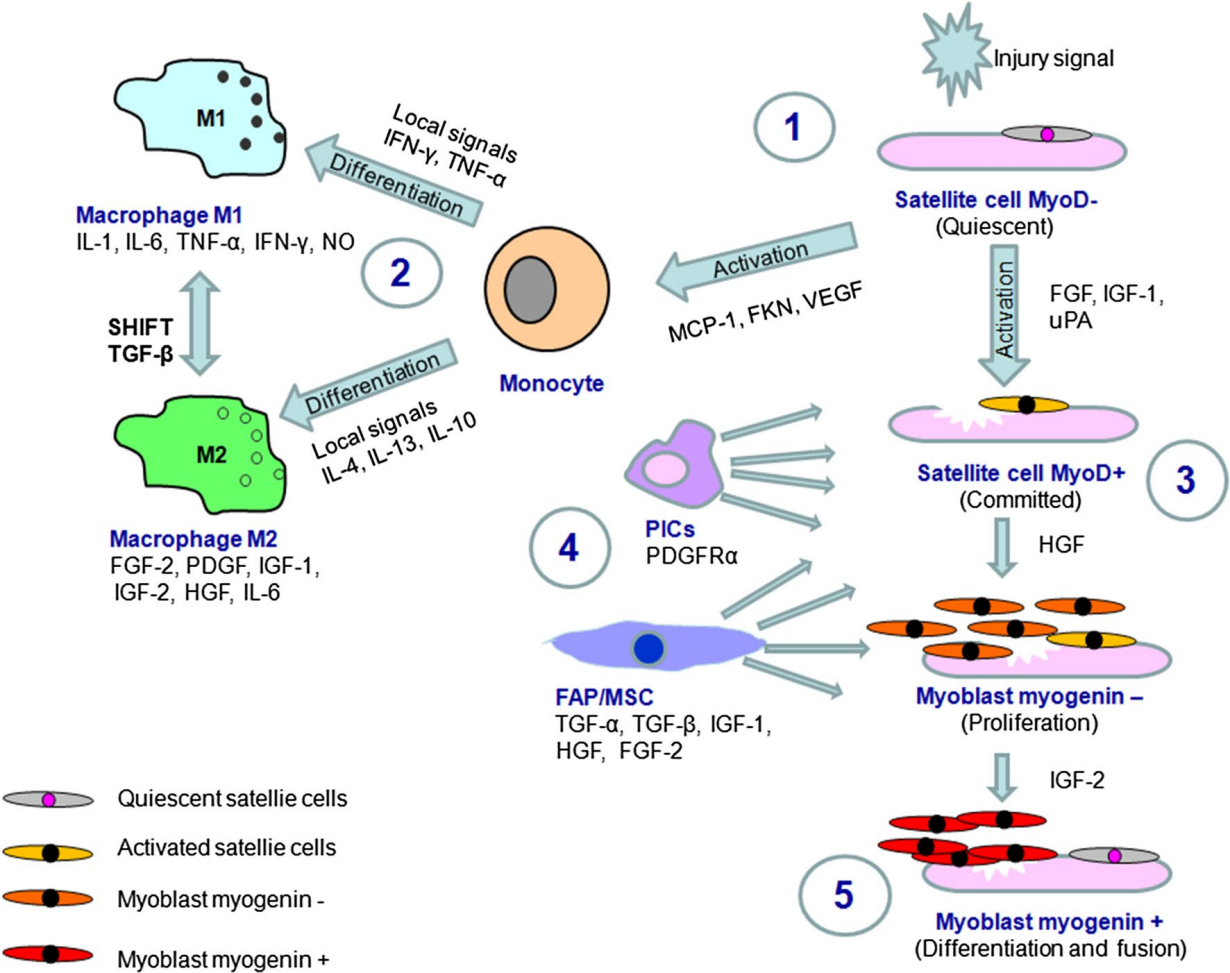
Activation and differentiation of muscle-resident satellite cells. Factors from local injury site activate quiescent satellite cells (1). Activated satellite cells and factors secreted by injured muscle attract monocyte into site of damage (2). Under environmental signal monocyte differentiates into M1 (pro-inflammatory) or M2 (supportive for muscle regeneration) macrophages (2). Activated satellite cells shift into differentiation cascade via committed MyoD^+^ cells (3) into myoblasts expressing myogenin. Non-satellite cells FAP/MSC and PICs secrete trophic factors into environment (4) supporting committed MyoD^+^ satellite cells activity. After proliferation and terminal differentiation myoblasts fuse to pre-existing injured myofibre or fuse one to another to form new myotubes, thus completing regenerative process (5). Quiescent satellite cell pool is renewed. FKN: fractalkine; MCP-1: monocyte chemotactic protein 1

*Satellite cells* are a well-known muscle-resident cell population involved in muscle growth and regeneration (Boonen and Post 2008; Judson et al. 2013; Srikuea et al. 2010; Yin et al. 2013). They are located in the specific muscle stem cell niche, between muscle fiber and the basal lamina, and they are naturally quiescent until an activation signal from the local environment is delivered. SCs participate in self-renewal and myogenesis after their activation. In the most common situations of muscle injury, SC activation is initiated by a signal delivered from myofibers in stress conditions. When SCs move out from the quiescence niche, they interact with stromal components that support development of their myogenic differentiation program and promote cell survival. Injured muscle fibers produce a number of growth factors including FGF, HGF and IGF, which are involved in the proliferation and differentiation of SCs (Boonen and Post 2008; Srikuea et al. 2010). Quiescent SCs naturally express the transcriptional factor Pax7 and most of them (but not all) express Pax3 as well as Myf5, but they not express MyoD or myogenin (Lagha et al. 2008) (Fig. 1). Moreover, quiescent SCs naturally express the fibroblast growth factor receptor gene *Fgfr4* and the myogenic fate determining gene *Myf5*, and both are controlled with Pax3/Pax7 family transcription factors (Lagha et al. 2008; Pannerec et al. 2013). An activation signal, directed under Pax3/Pax7 regulatory transcriptional control, leads to the activation and proliferation cascade and induces expression of muscle-specific transcriptional factors including MyoD, Myf5 and myogenin, which lead to proliferation of SCs, and are subsequently involved in differentiation of SCs into myoblasts (Lagha et al. 2008). Proliferating SCs are referred to as MPCs. The terminal differentiation process of MPCs is associated with downregulation of transcriptional factor Pax7 and with myogenin and MyoD expression. Damaged muscle also expresses SDF-1 (ligand for CXCR4) which reacts with the CXCR4 chemokine receptor present on the SC surface. Upregulation of SDF-1 on injured muscle facilitates SC migration into the site of injury (Yin et al. 2013). During the proliferation phase, committed SCs secrete chemokines and factors exhorting other adjacent cells to promote their survival and differentiation. The regeneration process, in addition, is supported by growth factors secreted by cells arriving from the SC niche. Activity of SCs is also regulated by their interactions with cells of myeloid origin including macrophages (Fig. 1), which constitute the stromal cell type present at the site of muscle injury (Boonen and Post 2008).

Regenerative-friendly macrophages of M2a and M2c phenotype usually reach the muscle about the second day after injury (Tidball and Villalta 2010). This is also the time when the SCs activate the myogenic program, migrate from their niche and increase in numbers in the proliferation process. There are several factors that allow activated SC migration into the site of injury. The process of migration is not too easy, because quiescent SCs are localized under the basal lamina and need to migrate to the injured site through the ECM. Remodeling of the ECM is facilitated by matrix metalloproteinase (MMP)-2 and MMP-9, which can be secreted by SCs and are upregulated during muscle regeneration (Boonen and Post 2008). Inhibition of MMP activity affects migration of muscle-derived stem cells in vitro (Bellayr et al. 2013). Moreover, migration of activated SC from their niche is also regulated by syndecan-3 expression in SCs as proved in studies on a syndecan-3 null mouse model (Pisconti et al. 2016). After migration and expansion near the site of injury, activated SCs are ready to form myoblasts—cells involved directly in the formation of myotubes or fusion with damaged myofibers. While approaching the phase of differentiation, SCs strongly express insulin-like growth factor binding protein-5, which makes factors from the IGF family able to bind. Especially the factor IGF-2 is known to provide strong signaling promoting SCs to enter the phase of myogenic differentiation (Boonen and Post 2008; Goldspink 2005; Harridge 2003). Newly formed, young myoblasts at first are myogenin negative—this prevents their differentiation into myofibers or fusion with the old ones, and allows an increase of the number of myoblasts. Then, when the number of myoblasts becomes correspondingly greater, there appears myogenin leading directly into myoblast differentiation (Yin et al. 2013). Later, in the phase when myogenic cells fuse to existing damaged fibers or fuse with one another to form myofibers de novo, IGF silences the process of cell proliferation and differentiation. Also, TGF-β modulates maturation of new fibers, influencing synthesis of collagen related with tendon. Long-term muscle integrity is permitted by the ability of SCs to return to quiescence to maintain the SC pool (Fig. 1).

In muscular dystrophies, SCs actively participate in muscle regeneration; however, each cell cycle shortens the telomeres of SCs, leading to cell senescence and a rapid decrease of the SC pool (Decary et al. 2000). In the DMD environment, SCs preserve regenerative capacity, but the dystrophic niche is unfavorable for efficient muscle regeneration, as proved in an mdx mouse model (Boldrin et al. 2015). Recent studies on muscle stem cells of mdx mouse documented that dystrophin is also expressed in activated SCs and controls the determination of SC polarity and asymmetric divisions. The authors suggested that impaired regeneration of dystrophic muscle in DMD patients is aggravated by intrinsic SC dysfunction and the consequent limited regenerative lifetime of dystrophin-deficient SCs (Dumont et al. 2015).

Therefore, biological properties of SCs of dystrophic muscle are insufficient to maintain the regenerative potential in the dystrophic environment, and employment of SCs from a healthy donor may take over the biological function of damaged SCs.

## Non-satellite Cells in Muscle Regeneration

SCs after activation need strong paracrine support from their niche, because without it they will not be able to survive. Data have shown that the majority (about 95%) of transplanted SCs die without anti-apoptotic signals, and that without pro-differentiation and pro-proliferative factors muscle regeneration will not be possible (Chazaud et al. 2003; Skuk and Tremblay 2000). Apart from SCs, a number of non-satellite stem cells including mesenchymal stem/stromal cells MSC, PW1^+^/PAX7^−^ interstitial cells, FAPs/MSCs, muscle SP cells and muscle resident pericytes are active and participate in the muscle regeneration process (Boppart et al. 2013). The myogenic potential of non-satellite progenitor cells was recognized in a cell population located in the muscle interstitium in the neonate (Mitchell et al. 2010). These cells revealed multilineage potential and belong to mesenchymal progenitor/stromal cells, as confirmed by the wide range of gene expression common to MSCs (Pannerec et al. 2013).

*Fibrocyte*/*adipocyte progenitors* (FAPs) are bipotent cells able to differentiate into fibroblasts and adipocytes in vitro—hence the term fibrocyte/adipocyte. Also, there is another term used to describe them, FAPs/MSCs, because in fact they are MPCs (mesenchymal progenitor cells)—bipotent progenitors obtained through one of the MSC maturation pathways (Faralli and Dilworth 2014; Joe et al. 2010). Under steady-state conditions, FAPs do not contribute directly to regeneration of muscle fibers. The direction of FAP differentiation can be regulated by the microenvironment, especially by upregulation of IL-6 and IGF-1, which may have a great pro-differentiate influence on SCs and myoblasts as documented in studies on a mouse model (Joe et al. 2010). The phenotype of FAPs, which may acquire a more myogenic than adipogenic fate, is recognized as Sca^+^/integrin^−^, but FAPs definitely are not involved in muscle regeneration by direct differentiation and forming myofibers (Joe et al. 2010; Judson et al. 2013). In the case of muscle injury, eosinophils infiltrating the injured area secrete IL-4 and IL-13, which activate FAPs and inhibit FAPs adipogenic conversion after muscle injury (Heredia et al. 2013). Activated FAPs have the ability to form a fibrotic muscle scaffold supportive in the muscle regeneration process (Faralli and Dilworth 2014; Joe et al. 2010).

However, the situation is not clear when the compensatory response of degenerating muscles is associated with formation of fibrotic scars and excessive fat infiltration (Serrano et al. 2011). The study revealed that FAPs in *mdx* mice might actually lead to fibrosis or fat deposition in muscle; furthermore they may contribute to diminution of myofiber contractility, retarding its metabolism. Paradoxically, FAPs can be involved in treatment of dystrophy as well (Mozzetta et al. 2013). The study revealed that treating FAPs of young *mdx* mice with trichostatin A (TSA), a member of HDACi (histone deacetylase inhibitors), can block their fibrotic and adipogenic differentiation, and promote myogenic fate, by changing the organization of chromatin structure (Saccone et al. 2014). RNA analysis showed a decrease of adipogenic genes and upregulation of myogenic genes in FAPs after TSA treatment (Mozzetta et al. 2013; Saccone et al. 2014). This pharmacological influence on FAPs can be applied for regeneration of dystrophic muscles and may prevent the deleterious effect associated with fibro/adipogenic changes of dystrophic muscles.

*PW1*^+^/*PAX7*^−^ *interstitial cells* Interstitial stem cells, positive for stress mediator PW1 expression and negative for transcriptional factor PAX7, called PICs, constitute an important muscle-resident stem cell population involved in perinatal skeletal muscle growth and during the adult muscle regeneration process (Mitchell et al. 2010).

These muscle interstitial cells are characterize by the expression of the muscle-specific progenitor marker CD34, and they are negative for endothelial markers, as proved by CD31 negative staining (Bosnakovski et al. 2008). Studies have shown that these cells contribute to regenerative myogenesis and SC generation, as documented in vitro when co-cultured with myoblasts or in vivo when transplanted into the regenerating muscle environment (Mitchell et al. 2010). PW1^+^ interstitial cells express a variety of genes common to MSCs (Oct3/4, Sox2, Nanog) (Cottle et al. 2017), and a subset of PICs expressing PDGF receptor (PDGFR)α overlap the cell surface expression and function of FAPs (Pannerec et al. 2013).

*Side population cells* These are muscle-resident progenitors located in the skeletal muscle interstitium next to blood vessels, which makes them distinguishable from bone marrow-derived SP cells and from SCs. They are a heterogeneous population of muscle-resident progenitors which contribute to both muscle and vascular regeneration (Asakura et al. 2002; Majka et al. 2003). Myogenic differentiation was induced in co-culture with primary myoblasts or through the induced expression of PAX7 or MyoD (Asakura et al. 2002). The majority of muscle SP cells express the endothelial marker CD31^+^, making them an attractive candidate to induce vasculogenesis, necessary for proper muscle regeneration. However, a fraction of muscle-origin SP cells, CD31^−^/CD45^−^, isolated from injured muscle, also express proangiogenic factors including angiopoietin-1 and VEGF, and factors associated with mesodermal/mesenchymal nature of cells, e.g. PDGFRα. Thus, studies on muscle-resident SP cells suggest that SP cells within the muscle constitute a sub-fraction of mesenchymal-like stem cells and/or pericytes, and both directly and indirectly contribute to muscle repair.

*Pericytes* Muscle resident pericytes are the next muscle progenitors of mesenchymal origin with a cell marker signature identical to MSCs (Birbrair et al. 2014; Caplan and Correa 2011; Crisan et al. 2008; Dellavalle et al. 2011; Traktuev et al. 2008). In fact, most pericytes are a quiescent type of MSCs residing on the surface of blood vessels and appear once in every 100 endothelial cells. Multipotential character of pericytes, sorted by CD146^+^, CD34^−^, CD45^−^ and CD56^−^ expression, was confirmed by their osteogenic, chondrogenic, adipogenic and myogenic potential (Caplan 2007). However, pericytes are heterogeneous, and the phenotype and biological activity of pericytes depend on their tissue localization. Differentiation potential of pericytes might be induced by the environment of cells surrounding them, so while residing on the surface of blood vessels penetrating (for example) smooth muscle, pericytes will gain inner potential to form smooth muscle, and preserve a lot of attributes characteristic for all pericytes independent of their tissue localization (Birbrair et al. 2014; Cappellari and Cossu 2013). Studies by Birbrair et al (2013), performed on double transgenic mice, documented that in the skeletal muscle there are two types of pericytes, type 1 (Nestin-GFP^−^/NG2-DsRed^+^) and type 2 (Nestin-GFP^+^/NG2-DsRed^+^), and only the latter is a marker allowing cells to enter the myogenic differentiation process. Moreover, type 2 vessel associated pericytes express the transcriptional factor PAX7 and in appropriate conditions can accomplish satellite cell position and function. In contrast, type 1 pericytes in skeletal muscle express PDGFRα, which is the major contributor to ectopic adipocyte formation in muscular dystrophies and in older adults (Birbrair et al. 2014). An in vitro study performed by Crisan et al. (2008) revealed that pericytes isolated from human tissues might have even greater myogenic potential than myoblasts.

However, in addition to their direct contribution to muscle tissue regeneration, pericytes have another very special function. When new blood vessel formation is necessary, for muscle regeneration or in ischemic conditions, pericytes play a significant role. Neoangiogenesis takes place in response to trophic factors such as VEGF, FGF-2, PDGF and PIGF (placental growth factor) secreted by myofibers, fibroblasts and inflammatory cells, which are essential for muscle survival in such conditions.

Unfortunately, pericytes, similarly to FAPs, are also suspected to cause fibrosis and fat deposition in dystrophic muscles and both of them express the PDGFRα receptor (Birbrair et al. 2014; Olson and Soriano 2009; Uezumi et al. 2011). Naturally, in normal conditions PDGFRα is an essential factor in many processes, including cell development and angiogenesis, but some studies have revealed that prolonged activation of this receptor, induced by mutation, may actually cause multiple organ fibrosis in the adult mouse body (Olson and Soriano 2009). The pathological contribution of mesenchymal progenitors, FAPs and pericytes, may be induced by TGF-β—a factor which stimulates expression of collagen I and III type and connective tissue growth factor (Uezumi et al. 2011).

Summarizing activity of muscle-resident non-satellite cells in muscle regeneration, it is important to note that they play dual roles: in healthy muscle they have an influence on SC differentiation and a significant function in myogenesis, but in unfavorable conditions, such as in muscular dystrophies, they contribute to fibrosis and adipose tissue accumulation. Therefore, manipulation of biological activity of non-satellite cells may support therapeutic strategies to treat muscular dystrophies.

## Combined Therapy with MSCs of BM Origin and SCs of Skeletal Muscle Origin for Potential Application for Muscular Dystrophy Treatment

Cellular therapies in muscular dystrophies are not a new idea. Studies on progression of muscular dystrophy documented that rapid occurrence of the dystrophic phenotype in the dystrophin/utrophin double knock-out mice model was associated with a rapid depletion of the functional MPCs. The authors suggests that preventing the depletion of the MPC pools could be a novel approach to delay the histopathologic feature associated with the skeletal muscles of DMD patients (Lu et al. 2014). Several studies have been performed to introduce different cellular therapies in muscular dystrophies; however, the rate of success has been limited (reviewed by Cossu and Sampaolesi 2007; Farini et al. 2009; Meng et al. 2011; Price et al. 2007). The cells most often applied for cellular therapies in muscular dystrophies are myoblasts, muscle precursor cells or stem cells with ability to differentiate into muscle cells. Early clinical studies, performed over 20 years ago, with adoptive transfer of myoblasts, isolated from skeletal muscle of healthy donors, seemed to show a promising strategy to restore skeletal muscle function; however, limited positive results were reported (Karpati et al. 1993; Law et al. 1993; Mendell et al. 1995; Miller et al. 1997; Skuk et al. 2004). These poor results using myoblast transfer may be explained by immunosuppression, an inadequate number of transplanted cells and limited distribution of transplanted cells, as myoblasts have limited migratory capability and limited proliferative potential (Mouly et al. 2005; Skuk et al. 2004). Moreover, allogeneic myoblast delivery may induce a strong immune response, in consequence leading to allograft rejection.

Promising results of cell-based therapy in DMD treatment were reported using the “high density injection” protocol for intramuscular cell delivery of muscle precursor cells from allogenic (sibling) healthy donors under a tacrolimus regimen (Skuk et al. 2006, 2007). Restoration of donor-derived dystrophin expression was observed in 27.5% of myofibres 1 month after cell transplantation, and 34.5% 18 months after intramuscular cell delivery; however, a significant improvement in strength was not observed. Success in local therapeutic cell delivery via intra-femoral arterial perfusion of skeletal muscle was also reported in studies using human induced pluripotent stem cells of myogenic origin in an immunodeficient mouse model NSG-mdx(4cv) for DMD (Matthias et al. 2015). Four weeks after intra-arterial cell delivery human cells were detected in the interstitial space of myofibers within the perfused muscle, and some of them fused with the recipient myofibers and expressed dystrophin. A clinical study on intra-arterial HLA-matched donor mesoangioblast transplantation in five DMD patients documented the presence of a low level of donor DNA in muscle biopsies in 4/5 patients and donor-derived dystrophin in one patient. A study showed that intra-arterial cell delivery is a feasible and relatively safe procedure, but functional improvement was not observed (Cossu et al. 2015). On the other hand, an experimental study showed that specific muscle-resident human dystrophin-positive mesoangioblasts from healthy donors co-cultured with dystrophin-negative myoblasts from DMD patients in vitro in a microengineered model of DMD resulted in cell fusion and functional differentiation of myotubes and dystrophin expression (Serena et al. 2016). Therapeutic potential of muscle-derived stem/progenitor cells of human origin was also tested on athymic mouse and rat models. Highly myogenic (CD34^−^/CD45^−^/CD29^+^) fraction revealed an active contribution to muscle fiber regeneration whereas multipotent stem cell (CD34^+^/CD45^−^) revealed multiple differentiation potential as confirmed by engraftment to the interstitium and differentiation into Schwann cells, perineurial/endoneurial cells, vascular endothelial cells and pericytes. Moreover, co-transplantation of both populations of cells, separately expanded, showed favorable results in skeletal muscle regeneration. Therefore, synergistic effect of co-transplantation of highly myogenic and multipotent stem cells (both of human muscle-origin) may be promising tool for muscle regeneration in autologous conditions in non-genetic muscular injuries (Tamaki et al. 2015).

The recognition of stem cell-myogenic precursors such as SCs, muscle-derived stem cells, SP cells, BM-derived stem cells, mesoangioblasts, muscle-derived CD133^+^ stem cells and pericytes seems to be a promising strategy for their application to overcome difficulties related to more differentiated myoblasts (Farini et al. 2009). Stem cell-myogenic precursors are more primitive than myoblasts and are able to proliferate and could be distributed through the blood vessels to the whole body musculature to treat severely affected patients (Farini et al. 2009; Peault et al. 2007). Moreover, some populations of myogenic precursors such as CD133^+^ cells or pericytes may be isolated not only from skeletal muscle but also from bone marrow and blood. Very recent studies on human myogenic cells introduced a population of human myogenic reserve cells (about 38.0%) which are not able to fuse in two-day culture in the differentiation medium, and are distinct from those which differentiate into myoblasts and fuse (about 62.0%). The human myogenic reserve cells generated in vitro significantly augmented the number of myogenic progenitor cells expressing PAX7, as compared to human myoblasts, after intramuscular transplantation in immunodeficient mice (Laumonier et al. 2017).

**Fig. 2:**
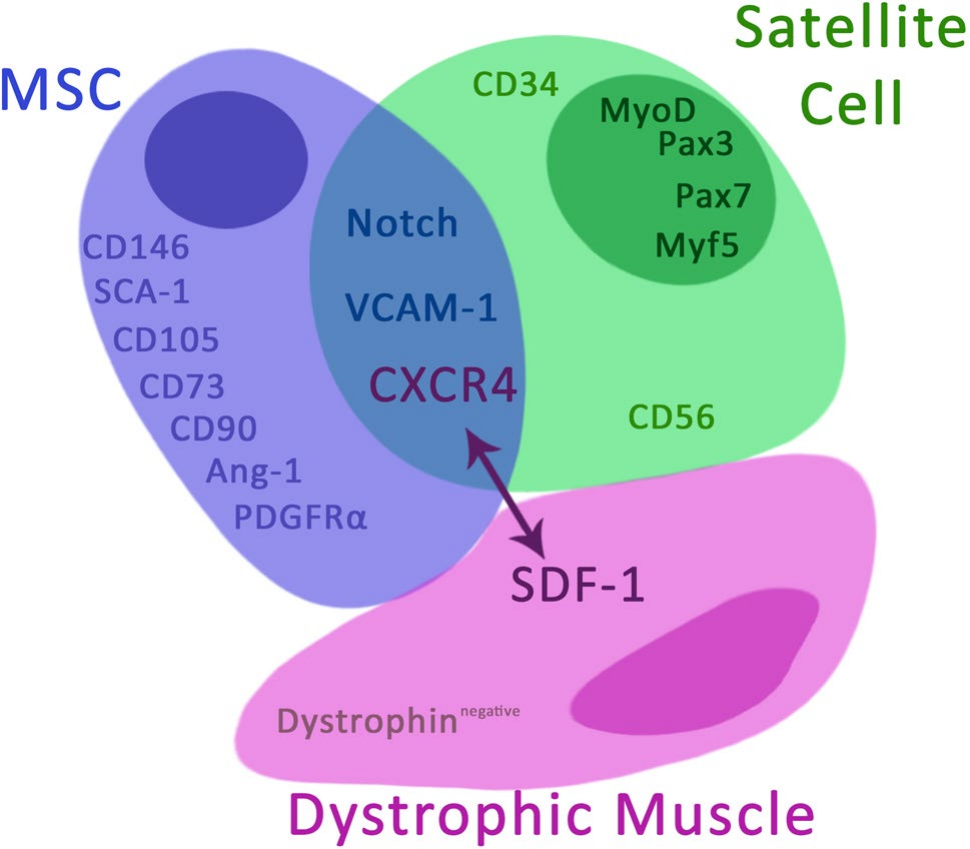
Hypothetical mechanism of dystrophic muscle regeneration by combined cellular therapy with MSC of bone marrow origin and muscle stem/progenitor cells. The local MSCs delivery into dystrophic muscles will create the microenvironment supporting homing of myogenic precursors and enhance tropism of stem cells of myogenic origin with the CXCR4^+^ expression to the injured muscle expressing SDF-1. Collaborative activities of MSC and satellite cells enhance regenerative potential of stem/progenitor cells of muscle-origin by direct contact and by secretion of trophic factors which influence on muscle stem/progenitor cells proliferation and myogenic differentiation

Based on the current knowledge and our own experience on the biology of MSCs of BM origin and stem/progenitor cells of skeletal muscle origin (Klimczak et al. 2016) we propose combined cellular therapy with MSCs of BM origin and muscle stem/progenitor cells for treatment of patients suffering from DMD. We hypothesize that both cell populations will fuse with damaged muscle cells and repopulate the muscle with dystrophin, improving muscle function (Fig. 2). Apart from stem/progenitor cells of muscle origin, also MSCs of BM origin have been shown to be able to participate in myogenesis as they are able to differentiate into mesodermal cells, including myoblasts (Bhagavati and Xu 2004; Fairclough et al. 2011). In addition, MSCs have pro-angiogenic potential and they might participate in blood vessel formation by direct differentiation into endothelial cells and/or as supporting niche cells for vascular (re-)generation, which is critical for proper muscle function (Lin and Lue 2013; Watt et al. 2013).

Moreover, immunosuppressive properties of MSCs may inhibit the inflammatory process at the site of stem cell delivery. Muscle degeneration in DMD is associated with chronic inflammation associated with active production of TNF-α by infiltrating M1 macrophages (Ichim et al. 2010). MSCs have the potential to convert inflammatory M1 type of macrophages (pro-inflammatory, anti-angiogenic and inhibitors of tissue growth) to M2 phenotype (anti-inflammatory, pro-remodeling and tissue healing) by secretion of IL-4 and IL-10, cytokines which support a shift from M1 to M2 type. This effect is required for skeletal muscle and neural healing and regeneration (Murphy et al. 2013; Rigamonti et al. 2014).

Muscle-derived stem/progenitor cells are heterogeneous population of muscle precursors with different phenotype depending on stage of differentiation. Most stem cell-myogenic precursors (about 70%), such as SP cells and SCs, are CD34^+^ cells which express VLA-4 (ligand for VCAM-1), and CXCR4, a chemokine receptor specific for SDF-1 (also named CXCL12) (Ichim et al. 2010; Perez et al. 2009). SDF-1 is upregulated in dystrophic muscle, whereas VCAM-1 is upregulated on the vessel endothelium in the dystrophic muscle environment. Experimental studies confirmed that intra-arterial delivery of myogenic precursors expressing CXCR4^+^ enhances their ability to extravagate into dystrophic muscle (Gavina et al. 2006). However, intravascular infusion of a large number of non-hematopoietic stem/progenitor cells of muscle origin will be risky for patients. Some studies suggest that a more therapeutically relevant method would be administration of MSCs directly into dystrophic muscle where they may contribute to formation of new muscle fibers and muscle neovascularization. In the dystrophin-deficient *mdx* mouse model, transplantation of primary human MSCs genetically modified with Pax3 into tibia muscle of *mdx* deficient mice resulted in donor-origin myofiber formation, but functional recovery was not achieved. The authors emphasized the lack of evidence that human MSC-Pax3 contributes to the satellite cell compartment in vivo. Fusion of donor cells with host myofibers rather than reprogramming of MSCs into myogenic progenitors may contribute to dystrophin-positive myofiber formation. Based on this observation, the authors suggest that multiple cell injections might be required to trigger functional recovery of dystrophic muscle (Gang et al. 2009). This proposal is in line with our hypothesis that MSCs alone or muscle progenitors alone are not able to restore dystrophin-deficient muscle function and local cell delivery may be a more effective method. Local MSC delivery into dystrophic muscles will create a microenvironment supporting homing of myogenic precursors and enhance tropism of stem cells of myogenic origin with CXCR4^+^ expression to the injured muscle expressing SDF-1 (Fig. 2). Studies have shown that signaling through CXCR4/SDF-1 interaction stimulates satellite cell migration (Ratajczak et al. 2003; Sherwood et al. 2004). However, in Sherwood and co-workers’ studies, functional heterogenicity between muscle-resident progenitor cells was introduced in a mouse model. Bone marrow-derived myofiber-associated cells do not form myogenic colonies when cultured alone, but some of them are able to generate myoblasts and myotubes when co-cultured with myogenic cells. They are non-hematopoietic cells and the authors suggest that these cells are MSCs of BM origin (Sherwood et al. 2004). These studies again confirmed our hypothesis that MSCs of BM origin can contribute to myogenic recovery when co-transplanted with muscle-resident progenitor cells.

Our hypothesis on the regenerative potential of MSCs of BM origin in muscular dystrophies is also supported by very recent studies by Maeda et al. (2017). The authors documented that transplantation of MSCs of BM origin into peritoneal cavities of a *mdx* mouse model strongly suppressed dystrophic pathology in diaphragms of *mdx* deficient mice, which resulted in significant lifespan extension.

Moreover, MSC plasticity will cause that in the vicinity of injured muscle these cells will differentiate into myoblasts producing dystrophin, which may enhance the regenerative effect on dystrophic muscles. This is supported by studies performed ex vivo on rat origin cells documenting that myogenic differentiation of MSCs is facilitated by co-culture with primary myoblasts stimulated by basic FGF and dexamethasone (Beier et al. 2011). Subsequent studies by the same research group in a rat model showed that myogenic differentiation of MSCs of BM origin upon mono- and co-cultivation with myoblasts in the presence of HGF and/or IGF-1 was successful on a biocompatible 3D nanofiber scaffold. However, HGF and/or IGF-1 stimulation was not essential for successful myogenic differentiation (Witt et al. 2017).

The studies described above clearly suggest that in contrast to transplantation of only myoblasts with limited migratory potential, simultaneous co-transplantation of MSCs of BM origin and myogenic stem/progenitor cells of skeletal muscle origin seems to be the most effective method for cellular therapies because they possess the ability to proliferate and expand, to fuse with dystrophic muscular cells, and to migrate to affected muscle.

These clinical and experimental results demonstrate that it is important to find a method to reconstruct functional muscles, severely affected by fat and fibrotic tissue, in order to restore strength either by local application of specific stem cell-myogenic precursors or by systemic cell delivery. Thus, the development of cell-based therapies for muscular dystrophies by the delivery of two populations of normal stem/progenitor cells (MSCs of BM origin and SCs with myogenic potential isolated from healthy skeletal muscle) would be a promising tool to treat muscular dystrophies. Biological properties of MSCs of BM origin and stem/progenitor cells of skeletal muscle origin justify combined therapy by using both cell populations because to date there is no alternative method to treat patients suffering from DMD.

## Concluding Remarks and Future Directions

Therapeutic approaches for DMD have extensively been developed in recent years. Unfortunately, most of the strategies such as gene therapy to replace the mutated gene or to repair the endogenous gene [exon skipping or skipping premature termination (PTC124)] are effective only for specific mutations or for nonsense mutations and cannot be applied to all DMD patients (Cossu and Sampaolesi 2007; Farini et al. 2009). Recent studies, using muscle-derived stem cells of DMD patients transduced with dystrophin constructs and transplanted into an immunodeficient mouse model of DMD, documented dystrophin production functional in vivo (Meng et al. 2016). After extensive experimental procedures and clinical trials, researchers proposed that the most promising strategies for the treatment of muscular dystrophies will be a combination of different approaches including stem cell therapy in combination with gene therapy [reviewed by (Crist 2017; Pini et al. 2017)].

The therapeutic effect of individually transplanted MSCs of BM origin or stem/progenitor cells of muscle origin is limited due to the complexity of muscular dystrophies and biological properties of stem/progenitor cells. Muscle-origin stem/progenitor cells are currently not applicable to treat muscular dystrophies due to difficulties to keep stemness ex vivo. We propose therefore combined therapy including two populations of stem/progenitor cells of bone marrow and muscle origin by local intramuscular co-transplantation. MSCs of BM origin may create a more favorable environment for muscle progenitor cells in dystrophic muscle by secretion of immunosuppressive cytokines and enhance the regenerative potential of stem/progenitor cells of muscle origin by direct contact, and by secretion of trophic factors which control muscle stem/progenitor cell proliferation and myogenic differentiation. Collaborative activities of MSCs and muscle stem/progenitor cells may influence further the changes in the dystrophic microenvironment. These changes concern different molecules, cells and structures that constitute the dynamic niche supporting the regenerative potential of stem/progenitor cells. Moreover, immunosuppressive properties of MSCs may reduce alloreactivity of muscle stem/progenitor cells in allogenic conditions.

Thus, characterization of a stem cell population effective for muscle regeneration, and timing, dosage and route of delivery may hold potential for treatment of genetic-related muscular dystrophies—but this remains a distant goal.

## Abbreviations

FGF: Fibroblast growth factor
DMD: Duchenne muscular dystrophy
ECM: Extracellular matrix
EGF: Epithelial growth factor
FAPs/MSCs: Fibro/adipogenic progenitors/mesenchymal stem cells
HGF: Hepatocyte growth factor
IGF: Insulin growth factor
MMP: Matrix metalloproteinase
MSC: Mesenchymal stem cell
MPC: Myogenic precursor cell
NO: Nitric oxide
PGE-2: Prostaglandin 2
PIC: PW1^+^/PAX7^−^ interstitial cell
PDGFR: Platelet-derived growth factor receptor
SC: Satellite cell
SP: Side population
SDF-1: Stromal-derived factor-1
uPA/uPAR: Urokinase-type plasminogen activator/urokinase-type plasminogen activator receptor
VEGF: Vascular endothelial growth factor
